# Resting‐state connectivity within and across neural circuits in anorexia nervosa

**DOI:** 10.1002/brb3.1205

**Published:** 2018-12-27

**Authors:** Blair Uniacke, Yun Wang, Dominik Biezonski, Tamara Sussman, Seonjoo Lee, Jonathan Posner, Joanna Steinglass

**Affiliations:** ^1^ Department of Psychiatry Columbia University Irving Medical Center New York New York; ^2^ Department of Psychiatry New York State Psychiatric Institute New York New York; ^3^ Rockefeller University New York New York; ^4^ Division of Mental Health Data Science New York State Psychiatric Institute New York New York; ^5^ Department of Biostatistics Columbia University Irving Medical Center New York New York

**Keywords:** anorexia nervosa, connectivity, executive control network, orbitofrontal cortex, salience network, striatum, triple network model

## Abstract

**Introduction:**

Obsessional thoughts and ritualized eating behaviors are characteristic of Anorexia Nervosa (AN), leading to the common suggestion that the illness shares neurobiology with obsessive–compulsive disorder (OCD). Resting‐state functional connectivity MRI (rs‐fcMRI) is a measure of functional neural architecture. This longitudinal study examined functional connectivity in AN within the limbic cortico‐striato‐thalamo‐cortical (CSTC) loop, as well as in the salience network, the default mode network, and the executive control network (components of the triple network model of psychopathology).

**Methods:**

Resting‐state functional connectivity MRI scans were collected in unmedicated female inpatients with AN (*n* = 25) and healthy controls (HC; *n* = 24). Individuals with AN were scanned before and after weight restoration and followed for one month after hospital discharge. HC were scanned twice over the same timeframe.

**Results:**

Using a seed‐based correlation approach, individuals with AN had increased connectivity within the limbic CSTC loop when underweight, only. There was no significant association between limbic CSTC connectivity and obsessive–compulsive symptoms or prognosis. Exploratory analyses of functional network connectivity within the triple network model showed reduced connectivity between the salience network and left executive control network among AN relative to HC. These abnormalities persisted following weight restoration.

**Conclusions:**

The CSTC findings suggest that the neural underpinnings of obsessive–compulsive symptoms may differ from those of OCD. The inter‐network abnormalities warrant examination in relation to illness‐specific behaviors, namely abnormal eating behavior. This longitudinal study highlights the complexity of the neural underpinnings of AN.

## 1 INTRODUCTION

Anorexia nervosa (AN) is a serious disorder with one of the highest mortality rates of any psychiatric illness (Arcelus, Mitchell, Wales, & Nielsen, 2011). It is characterized by fear of weight gain, preoccupation with body shape and weight, and severe restriction of food intake leading to significantly low body weight (American Psychiatric Association, 2013). AN most commonly emerges during adolescence (Herpertz‐Dahlmann, 2015; Swanson, Crow, Grange, Swendsen, & Merikangas, 2011) and approximately half of affected individuals develop chronic illness (Lock et al., 2010). Preoccupations with food, weight and shape, coupled with ritualized eating, and compensatory behaviors are prominent features of AN (Heebink, Sunday, & Halmi, 1995; Sunday, Halmi, & Einhorn, 1995). Among adolescents with AN, obsessive–compulsive symptomatology has been associated with poor treatment outcome (Råstam, Gillberg, & Wentz, 2003) and eating‐related obsessions have been shown to be a moderator of treatment outcome (Le Grange et al., 2012). These symptoms, together with high rates of comorbidity with obsessive–compulsive disorder (OCD; Anderluh, Tchanturia, Rabe‐Hesketh, & Treasure, 2003; Bastiani et al., 1996; Bulik, Sullivan, Fear, & Joyce, 1997; Kaye, Bulik, Thornton, Barbarich, & Masters, 2004), increased co‐occurrence of AN and OCD in family studies (Strober, Freeman, Lampert, & Diamond, 2007), and evidence of a high genetic correlation between the two disorders (Yilmaz et al., 2018) have led many in the field to consider whether AN and OCD share an underlying pathophysiology. This study probes these similarities by examining the neural underpinnings of obsessive–compulsive symptoms in AN.

Resting‐state functional connectivity magnetic resonance imaging (rs‐fcMRI) is a useful tool to study both specific neural circuits as well as large‐scale neural networks implicated in psychiatric disease. Rs‐fcMRI measures spontaneous fluctuations in blood oxygen level‐dependent (BOLD) signal. It is used to investigate the functional architecture of the brain at rest (Lee, Smyser, & Shimony, 2013). In AN, a number of rs‐fcMRI studies have demonstrated evidence of disturbed functional connectivity in brain regions associated with cortico‐striato‐thalamo‐cortical (CSTC) circuitry which have also been implicated in OCD (Biezonski, Cha, Steinglass, & Posner, 2016; Cha et al., 2016; Ehrlich et al., 2015; Haynos et al., 2018). Using network‐based statistics (NBS), Ehrlich et  al identified reduced functional connectivity in a thalamo‐insular subnetwork among underweight adolescents and young adults with AN relative to healthy control participants (HC) (Ehrlich et al., 2015). Another study measuring thalamo‐frontal functional connectivity in acutely ill individuals with AN relative to HC found evidence of altered connectivity between the thalamus and both the dorsal and anterior prefrontal cortices which were associated with impairments in tasks measuring cognitive control and working memory, respectively (Biezonski et al., 2016). Haynos et al. (2018) used a seed‐based approach to investigate ventral and dorsal frontostriatal circuits and found evidence of reduced functional connectivity in both circuits among underweight individuals with restricting type AN relative to HC. Cha et al. (2016) found increased left‐sided nucleus accumbens (NAcc)–medial orbitofrontal cortex (mOFC) connectivity among underweight AN relative to HC. Though both of these studies identified altered connectivity in anatomically similar circuitry, their findings diverged in the direction of the effect (hypo‐ vs. hyperconnectivity).

In OCD, there is strong evidence that disturbances in corticolimbic circuitry, specifically within the limbic CSTC loop, are involved in the disorder's pathophysiology (Harrison et al., 2013; Maia, Cooney, & Peterson, 2008; Saxena & Rauch, 2000; Vaghi et al., 2017). For example, a relationship between altered limbic CSTC functional connectivity and obsessive–compulsive symptomatology was supported by the finding that reduced left‐sided NAcc‐mOFC resting‐state connectivity was associated with obsessive–compulsive symptom severity among unmedicated adults with OCD (Posner et al., 2014). Taken together, these results suggest that corticolimbic functional connectivity may be important to understanding the neurobiology underlying obsessive–compulsive‐like symptoms in AN. It may be that, akin to OCD, limbic CSTC abnormalities underlie the obsessional thoughts and ritualized behavior characteristic of AN (Steinglass & Walsh, 2006).

Brain connectivity can be examined between specific neural regions, or more broadly through functional network connectivity (FNC) analysis, which measures the correlation of BOLD signal between large‐scale resting‐state networks. FNC is used to investigate network‐level hypotheses (i.e., the functional architecture between networks). One such model, the triple network model of psychopathology, proposes that dysfunction within and between three resting‐state networks associated with attention and cognitive control results in the development of psychopathology (Menon, 2011): the salience network (SN; nodes include the dorsal anterior cingulate cortex; dACC; and anterior insular cortices), the default mode network (DMN; nodes include the posterior cingulate cortex, medial prefrontal cortex, and lateral parietal lobules), and the executive control network (ECN; nodes include the dorsolateral prefrontal cortex; dlPFC; and lateral posterior parietal cortex). More specifically, the triple network model proposes that the SN, which is involved in detecting and filtering internal and external stimuli (Menon, 2011), is responsible for appropriately engaging the ECN to attend to task‐related external stimuli (Seeley et al., 2007) and disengaging the DMN, which is activated during internally oriented, self‐referential cognitive processes (Greicius, Krasnow, Reiss, & Menon, 2003; Greicius & Menon, 2004). In this model, the SN is thought to act as the connector, or central hub, between the DMN and ECN. Psychopathology is hypothesized to arise as a consequence of impaired connectivity within and across these three networks.

This model has gained support over the past decade as a number of studies across neuropsychiatric disorders have found evidence of disturbed functional connectivity between the SN, DMN, and ECN, including schizophrenia (Manoliu et al., 2013), depression (Zheng et al., 2015), ADHD (Cai, Chen, Szegletes, Supekar, & Menon, 2018), PTSD (Liu et al., 2017), dementia (Chand, Wu, Hajjar, & Qiu, 2017), addiction disorders (Zhang et al., 2017), and OCD (Posner et al., 2017; Stern, Fitzgerald, Welsh, Abelson, & Taylor, 2012). Recent work in OCD has also shown that disturbances in triple network and CSTC loop connectivity overlap in frontoparietal brain regions (Gürsel, Avram, Sorg, Brandl, & Koch, 2018), thereby integrating the triple network model with well‐established models of psychopathology.

Altered connectivity within and between the three networks has been identified in AN. Abnormal connectivity within the DMN has been found using whole‐brain independent components analysis (ICA) among acutely ill individuals with AN (Boehm et al., 2014; McFadden, Tregellas, Shott, & Frank, 2014) and individuals recovered from AN (Cowdrey, Filippini, Park, Smith, & McCabe, 2014). Altered connectivity has also been identified within the SN in underweight and recovered participants with AN using ICA (McFadden et al., 2014) and a seed‐based approach (Lee et al., 2014). Gaudio et al. (2015) identified decreased connectivity in the ECN in adolescents with new‐onset illness, while others have found abnormal connectivity within the Frontoparietal Network (FPN; analogous to the ECN) in ill and recovered participants with AN (Boehm et al., 2014, 2016). On the other hand, several studies have not found significant differences in the networks implicated in the triple network model in underweight individuals with AN (Boehm et al., 2016; Scaife, Godier, Filippini, Harmer, & Park, 2017) or participants recovered from AN relative to HC (Boehm et al., 2014; Phillipou et al., 2016; Scaife et al., 2017). One study that used FNC to measure cross‐network connectivity of these systems found no difference in network interactions between the SN, DMN, and FPN among women recovered from AN and HC (Boehm et al., 2016). Cross‐network interactions between the SN, DMN, and ECN have not been studied in acutely ill individuals with AN.

In this longitudinal study, we used rs‐fcMRI to measure functional connectivity in corticolimbic neural circuitry in adolescents and young adults with AN relative to age‐matched healthy peers. To better characterize neural mechanisms relevant to persistence of illness, participants were examined before and after weight restoration treatment. Building from existing data showing increased limbic CSTC connectivity among underweight adolescents and adults with AN (Cha et al., 2016) and evidence of an association between obsessive–compulsive symptoms and limbic CSTC functional connectivity in individuals with OCD (Posner et al., 2014), we hypothesized that NAcc–mOFC functional connectivity would (a) be increased among underweight AN relative to HC, (b) be associated with greater obsessive–compulsive symptoms in AN, and (c) partially normalize following weight restoration, with a concomitant reduction in obsessive–compulsive symptoms. We examined limbic CSTC functional connectivity bilaterally, though our a priori hypotheses were based on prior studies that found evidence of altered NAcc‐OFC rsFC in the left hemisphere only (Cha et al., 2016; Posner et al., 2014). Secondarily, because studies in OCD have associated obsessive–compulsive symptoms with altered interactions between the SN, DMN, and ECN, we explored broader network connectivity between these networks using FNC before and after weight restoration among AN relative to HC.

## 2 METHODS

### 2.1 Participants

Participants were individuals with AN and HC who presented to the Columbia Center for Eating Disorders (Table 1). Eligible patients were between 14 and 26 years old, met DSM‐5 criteria (American Psychiatric Association, 2013) for AN, restricting (AN‐R, *n* = 14) or binge‐purge (AN‐BP, *n* = 11) subtype, and were receiving inpatient treatment at the New York State Psychiatric Institute (NYSPI). Individuals were excluded if they had a history of a neurological or psychotic disorder, met criteria for OCD or a current substance use disorder, or were at imminent risk of suicide. Anxiety and depressive disorders were not exclusionary, as these disorders commonly present with AN (Hudson, Hiripi, Pope, & Kessler, 2007). HC were group‐matched for age and sex and were included if they had a BMI in the normal range (18–25 kg/m^2^), had no significant medical illness, and had no current or past psychiatric illnesses. Exclusion criteria for MRI included current use of psychotropic medication and pregnancy. In total, 25 individuals with AN and 24 HC participants were included in neuroimaging analyses.

**Table 1:** Demographics and clinical characteristics for all enrolled participants

	Time 1				Time 2			
	HC (*n* = 28)	AN (*n* = 25)	*t*_51_	*p*	HC (*n* = 25)	AN (*n* = 25)	*t*_48_	*p*
	Mean ± *SD*	Mean ± *SD*			Mean ± *SD*	Mean ± *SD*		
Age (y)	19.4 ± 2.9	19.1 ± 3.5	0.4	0.72				
BMI (kg/m^2^)	21.1 ± 1.6	16.5 ± 2.0	9.3	<0.001	21.0 ± 1.8	20.8 ± 1.1	0.5	0.6
Dur. Ill (yr)		3.59 ± 2.8						
EDE	0.2 ± 0.1	3.0 ± 1.1	−13.0	<0.001	n/a	2.1 ± 1.0	n/a	n/a
STAI(T)	28.2 ± 6.3	58.2 ± 13.4	−10.6	<0.001	30.4 ± 7.0	54.1 ± 13.2	−7.5	<0.001
BDI	1.1 ± 1.6	23.4 ± 11.5	−10.1	<0.001	1.1 ± 1.4	15.4 ± 10.3	−6.3	<0.001
YBC‐EDS	0.2 ± 0.7	19.7 ± 7.7	−12.8	<0.001	n/a	13.5 ± 6.6	n/a	n/a
OCI‐R	4.9 ± 5.2	19.4 ± 13.1	−5.4	<0.001	5.2 ± 5.2	15.2 ± 12.8	−3.5	0.001
RRS	32.9 ± 10.1	52.7 ± 12.2	−6.3	<0.001	33.0 ± 11.5	48.7 ± 16.6	−3.8	<0.001

Note

At time 1, EDE data are missing for 1 HC. At time 2, EDE data are missing for 1 AN. At time 1, STAI(T) data are missing for 1 AN. At time 2, STAI(T) data are missing for 3 HC and 1 AN. At time 1, BDI data are missing for 1 AN. At time 2, BDI data are missing for 4 HC and 3 AN. At Time 1, YBC‐EDS data are missing for 2 HC. At time 2, YBC‐EDS data are missing for 1 AN. At Time 2, OCI‐R data are missing for 1 HC. At Time 1, RRS data are missing for 2 HC and 1 AN. At Time 2, RRS data are missing for 1 HC.

AN: anorexia nervosa; BDI: Beck Depression Inventory; BMI: body mass index; Dur. Ill: duration of illness; EDE: Eating Disorder Examination; HC: healthy control subjects; OCI‐R: Obsessive–Compulsive Inventory‐Revised; RRS: Ruminative Responses Scale; STAI(T): State‐Trait Anxiety Inventory; YBC‐EDS: Yale‐Brown‐Cornell Eating Disorder Scale.

This study was approved by the New York State Psychiatric Institute Institutional Review Board. Following complete description of the study to participants, adult participants provided written informed consent, and adolescents provided written assent with parental consent.

### 2.2 Study procedures

Resting‐state functional connectivity MRI scans and clinical assessments were conducted at two time points. For individuals with AN, study procedures were completed within one week of hospital admission and again after weight restoration to a BMI of at least 19.5 kg/m^2^. Time between sessions was group‐matched for AN and HC (AN mean ± *SD = *57.1 ± 21.2 days; HC mean ± *SD* = 59.6 ± 29.8 days; *t*(48) = 0.33, *p* = 0.74).

### 2.3 Clinical assessments

Psychiatric diagnoses were established via the Semi‐Structured Interview for DSM‐IV (SCID) (Spitzer & Williams, 1988) and clinical interview. The diagnosis of AN was made according to DSM‐5 criteria (American Psychiatric Association, 2013). The Eating Disorders Examination semi‐structured interview (EDE) (Cooper, Cooper, & Fairburn, 1989) was administered to assess eating disorder severity. Estimated IQ was assessed using the Wechsler Test of Adult Reading (WTAR) (Green et al., 2008). Self‐reported depression and anxiety were assessed via Beck Depression Inventory‐II (BDI‐II) (Beck, Steer, Ball, & Ranieri, 1996) and State‐Trait Anxiety Inventory (STAI) (Spielberger, Gorsuch, & Lushene, 1970), respectively.

Obsessive–compulsive symptoms were assessed in two ways: (a) the Obsessive–Compulsive Inventory‐Revised (OCI‐R) (Foa et al., 2002), which measures general obsessive–compulsive symptom severity and (b) the Yale‐Brown‐Cornell Eating Disorders Scale (YBC‐EDS), which measures eating disorder‐related rituals and preoccupations (Sunday et al., 1995). The post‐treatment YBC‐EDS was administered by phone one week after hospital discharge. As an additional measure of internal preoccupation and repetitive thinking, participants completed the Ruminative Responses Scale, a 22‐item self‐report instrument (Treynor, Gonzalez, & Nolen‐Hoeksema, 2003).

Height and weight were obtained on a beam balance scale (Detecto, Webb, MO) on the day of testing. Patients with AN were contacted during the month after discharge to assess weight trajectory. As weight maintenance in the first four weeks after weight restoration treatment is one of the few variables that predict longer term course (Kaplan et al., 2009), weekly weights for the first four weeks following discharge were obtained and verified by a clinician whenever possible (18 out of 23 participants). Weight slope was defined as the average change in weight per week over the first 28 days following hospital discharge and was calculated using all available weights (Kaplan et al., 2009).

### 2.4 MRI acquisition

Images were acquired on a GE Signa 3T whole body scanner with a 32­channel head coil. First, a 3­Plane Localizer and T1­Weighted Anatomical scans (TR = 7.23, TE = 2.78, flip angle = 11°, FOV = 23 cm, slice thickness = 0.9 mm, voxel size = 0.9 × 0.9 × 0.9 mm^3^) were acquired. Participants were then instructed to close their eyes and let their minds wander freely while rs‐fcMRI data were collected using oblique echo‐planar images (TR = 2,000 ms, TE = 30 ms, single excitation per image, slice thickness = 3.5 mm, no gaps, FOV = 24 cm) with 3.75 × 3.75 × 3.5 mm^3^ resolution and whole‐brain coverage. Two 5‐min resting‐state scans (155 volumes) were obtained for each participant at each time point. The same imaging procedures were used for pre‐ and post‐weight restoration scans.

### 2.5 MRI analysis: seed‐based connectivity

To test NAcc–OFC resting‐state functional connectivity, we used a seed‐based methodology similar to the approach by Cha et al. (2016). Specifically, standard image preprocessing was performed using SPM 12 and the CONN toolbox version 17.b for functional connectivity analysis. Slice timing and spatial realignment were applied. Images were then coregistered with a high‐resolution anatomical scan, normalized into the Montreal Neurological Institute space, and resampled at 2 mm. Images were smoothed with a Gaussian kernel of 8 mm FWHM (full width at half maximum). To minimize the influence of non‐neural contributors to the fMRI signal, the BOLD time series was regressed against (a) five orthogonal time series extracted from white matter and cerebrospinal fluid separately using CompCor (component‐based noise correction) methods (Behzadi, Restom, Liau, & Liu, 2007) and (b) 12 motion‐related regressors (six estimated motion parameters plus their 1st‐order derivatives from the rigid realignment preprocessing step in SPM). BOLD signal was also “scrubbed” by identifying and removing volumes (and the adjacent, neighboring volumes) with framewise displacement (FD) > 0.5 mm or global signal intensity changes > 3 *SD* estimated using the artifact detection tool (ART) (www.nitrc.org/projects/artifact_detect). Although no runs met this criterion, a threshold of >20% scrubbed volumes was used to exclude runs from subsequent analyses. Other signal processing in the pipeline included temporal band‐pass filtering (0.008–0.09 Hz) and linear detrending. Head motion was comparable across groups (Supporting information Figure S1).

Seed‐based correlation analysis was used to investigate connectivity between the NAcc and OFC bilaterally. The NAcc seed was predefined from FSL Harvard‐Oxford Atlas maximum‐likelihood subcortical atlas (HarvardOxford‐sub‐maxprob‐thr25‐2 mm.nii). Connectivity maps were generated by calculating the Pearson correlation between the time series within the NAcc seed and the time series for all other voxels in the brain; Pearson correlations were then Fisher r‐to‐z transformed to allow for group‐wise comparisons. For hypothesis testing, mean connection strength between the NAcc and the mOFC, as defined by a published atlas (http://www.gin.cnrs.fr/en/tools/aal-aal2/), was extracted. Whole‐brain connectivity maps were used in exploratory analyses.

### 2.6 MRI analysis: functional network connectivity (FNC)

Additional exploratory analyses examined FNC, using independent component analysis (ICA) optimized for the characterization of broad neural networks (Laird et al., 2011; Smith et al., 2009). Specifically, fMRI data preprocessing was completed using FEAT (FMRI Expert Analysis Tool) version 6. Preprocessing procedures included motion correction, nonbrain removal, spatial smoothing with a Gaussian kernel of FWHM 8 mm, and high‐pass (0.01 Hz) temporal filtering (Woolrich, Ripley, Brady, & Smith, 2001). Four networks of interest (i.e., SN, DMN, left and right ECN) were identified using an automated and previously validated ICA‐based approach: ICA + FIX (FMRIB's ICA‐based Xnoiseifier) (Griffanti et al., 2014; Salimi‐Khorshidi et al., 2014).

Independent components analysis + FIX was conducted by randomly selecting 20 study participants for group ICA, and then manually identifying components as either “good” (i.e., indexing resting networks) or “bad/noise” (i.e., indexing noise/artifacts) following published procedures (Griffanti et al., 2017). A classifier was then trained using FIX to automatically identify and regress out noise components for the remaining resting fMRI datasets, yielding a denoised resting fMRI dataset for subsequent analyses. Lastly, to determine the effectiveness of ICA‐FIX in denoising this rs‐fcMRI dataset, head motion parameters derived from this dataset after ICA‐FIX denoising were compared to another denoising technique (Supporting information Figure S2). Group ICA was applied to the denoised rs‐fcMRI data. The number of components was set at 30, and the decomposition approach was set as multisession temporal concatenation. The group ICA spatial maps were converted to Z score maps, and the DMN, SN, and ECN (left and right separately) were first identified through visual inspection of the components derived from group ICA. Next, we calculated the anatomical overlap between these components selected by visual inspection and published functional maps of resting‐state networks. We confirmed that the selected components had greater overlap with the DMN, SN, and ECN relative to other resting‐state networks with three measurements (Dice, Jacquard, and PearsonsSCC) (Supporting information Table S3). Lastly, using dual regression, we extracted subject‐specific time series for each of the three components. To measure cross‐network functional connectivity between the three networks included in the triple network model, we calculated the correlation coefficients (CC) between the SN and ECN, the SN and DMN, and the DMN and ECN (CC_SN, ECN_, CC_SN, DMN, _CC_DMN,ECN_) using the extracted time series for each component.

### 2.7 Statistical analyses

Demographic and clinical characteristics were compared between diagnostic groups (AN vs. HC) for continuous variables (age, BMI, estimated IQ, STAI‐T, and BDI) using independent samples *t* tests. Eating disorder symptoms, mood, anxiety, and obsessive–compulsive symptoms before and after weight restoration were examined with repeated‐measures ANOVA (AN vs. HC). Associations between post‐weight restoration (Time 2) clinical measures and weight slope after discharge were assessed using Pearson's correlation. All statistical analyses for clinical data were performed using SPSS 24.0 with an alpha level of 0.05.

For the *seed‐based connectivity analysis*, hypothesis testing was conducted with a general linear model (GLM) with NAcc–mOFC as the dependent variable, Group (AN vs. HC) as the between‐subject variable, Time (pre‐ and post‐weight restoration) as the within‐subjects variable, and nuisance covariates (see section on “Confounding Variables”). Exploratory analyses used the same GLM but examined whole‐brain connectivity differences between individuals with AN and HC using the left and right NAcc seeds, and correcting for multiple comparisons with FDR_corrected_ < 0.05 (Nichols & Holmes, 2002). For the *FNC analysis*, group differences in cross‐network connectivity were analyzed using a GLM with the correlation coefficient representing SN‐ECN/SN‐DMN/DMN‐ECN functional connectivity serving as the dependent variable, Group (AN vs. HC) as the between‐subject variable, Time (pre‐ and post‐weight restoration as the within‐subjects variable, and nuisance covariates (see section on “Confounding Variables”). All voxel‐based second level analysis was done in CONN, and summary connectivity measures were performed using SPSS 24.0.

#### 2.7.1 Confounding variables

In each statistical analysis, we tested a full model with nuisance covariates, including demeaned age and estimated IQ. As standardized norms for the WTAR are not available for the 6 individuals under the age of 16, demeaned WTAR raw scores were used as the covariate. In a sensitivity analysis, the six participants under the age of 16 years were removed from the neuroimaging analyses, which did not change the pattern of results. We performed an additional GLM (Bonferroni corrected for multiple comparisons) to measure the effect of AN subtype (AN‐R vs. AN‐BP) on connectivity differences, with connectivity strength as the dependent variable, Group (AN‐R, AN‐BP, HC) as the between‐subject variable, Time (pre‐ and post‐weight restoration) as the within‐subjects variable, and demeaned age and WTAR raw score as covariates.

#### 2.7.2 Clinical correlates

Partial correlations were performed to measure associations between fMRI results and measures of obsessive–compulsive symptomatology and weight slope, controlling for age and estimated IQ (demeaned). The significance and confidence level was set at α = 0.05 (95% confidence interval).

## 3 RESULTS

Demographic and clinical characteristics of all enrolled participants are presented in Table 1. Six individuals with AN had at least one comorbid anxiety disorder (Generalized Anxiety Disorder, Post‐Traumatic Stress Disorder or Agoraphobia without panic disorder). No participant with AN met criteria for a comorbid depressive disorder. Participants included in neuroimaging analyses were matched for age (HC mean ± *SD *18.9 ± 2.7 years; AN mean ± *SD *19.1 ± 3.5 years; *t*(47) = −0.18, *p* = 0.86) and estimated IQ (HC mean ± *SD* = 108 ± 8.9; AN mean ± *SD* = 103 ± 10.2; *t*(40) = 1.71, *p* = 0.10).

Anorexia nervosa had significantly higher scores on baseline measures of eating disorder severity, anxiety, and depressive symptoms (Table 1). Among individuals with AN, there was a significant improvement on the EDE (Time: *F*[1,23] = 27.3, *p* < 0.001), BDI (Time: *F*[1,20] = 7.5, *p* = 0.01), and YBC‐EDS (Time: *F*[1,23] = 11.7, *p = *0.002) with weight restoration. Trait anxiety did not change with treatment (Time: *F*[1,22] = 2.7, *p* = 0.11). Scores on the OCI‐R were significantly higher among individuals with AN compared with HC at both time points (Group: *F*[1,47] = 23.6, *p < *0.001), Time: *F*[1,47] = 2.0, *p = *0.16), with a trend toward improvement among AN (Time* group (*F*[1,47] = 3.7, *p = *0.06). Rumination scores were also significantly higher among AN relative to HC (Group: *F*[1,44] = 25.6, *p < *0.001), with no effect of time (*F*[1,44] = 1.3, *p = *0.26) and no interaction between time and group (*F*[1,44] = 2.2, *p = *0.15). Associations between post‐weight restoration (Time 2) clinical assessments and rate of weight loss following discharge indicate that those individuals who were more symptomatic at Time 2 lost more weight in the first month following hospitalization: BDI (*r *= −0.58, *p = *0.01), OCI‐R (*r *= −0.50, *p = *0.02), YBC‐EDS (*r* = −0.53, *p = *0.01), and RRS (*r* = −0.42, *p* = 0.06).

### 3.1 MRI results: Seed‐based connectivity

**Figure 1:**
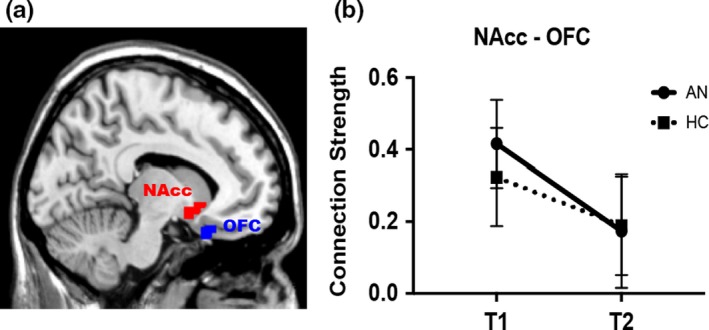
Individuals with anorexia nervosa (AN) show increased connectivity in the limbic cortico‐striato‐thalamo‐cortical loop during the underweight state. (a) Sagittal image showing the left nucleus accumbens (NAcc) and left medial orbitofrontal cortex (mOFC) region of interest. (b) Connection strength between the NAcc and left medial OFC before (T1) and after (T2) weight restoration treatment in participants with AN and healthy controls (HC) at matched time points. Connectivity strength was significantly greater in AN relative to HC at Time 1 (*F*[1,45] = 7.79, *p = *0.01) with no group difference at Time 2

Left NAcc–left mOFC connectivity showed a significant effect of group (*F*[1,45] = 7.7, *p = *0.01), a significant effect of time (*F*[1,45] = 62.6, *p < *0.001), and a trend toward an interaction between time and group (*F*[1,45] = 2.54, *p = *0.12) (Figure 1). There were no significant group differences in right NAcc‐mOFC connectivity strength. Post hoc analysis indicated increased left NAcc–left mOFC connectivity in the AN group relative to HC at Time 1 (*F*[1,45] = 7.79, *p = *0.01) with no difference at Time 2 (*F*[1,45] = 0.99, *p = *0.32). Secondary analysis of the effect of subtype on resting‐state connectivity showed increased left NAcc–left mOFC connectivity in the AN‐BP group relative to HC (Bonferroni corrected *p = *0.02), and no significant difference between AN‐BP and AN‐R (Bonferroni corrected *p = *0.17) or between AN‐R and HC (Bonferroni corrected *p = *1.0) at Time 1. The same patterns were observed at Time 2, but group differences were not significant (Bonferroni corrected *p*'s > 0.22). There were no significant associations between left NAcc–left mOFC connectivity and obsessive–compulsive symptoms among AN (Supporting information Table S1). Whole‐brain exploratory analyses yielded nonsignificant group effects.

### 3.2 MRI results: functional network connectivity (FNC)

**Figure 2:**
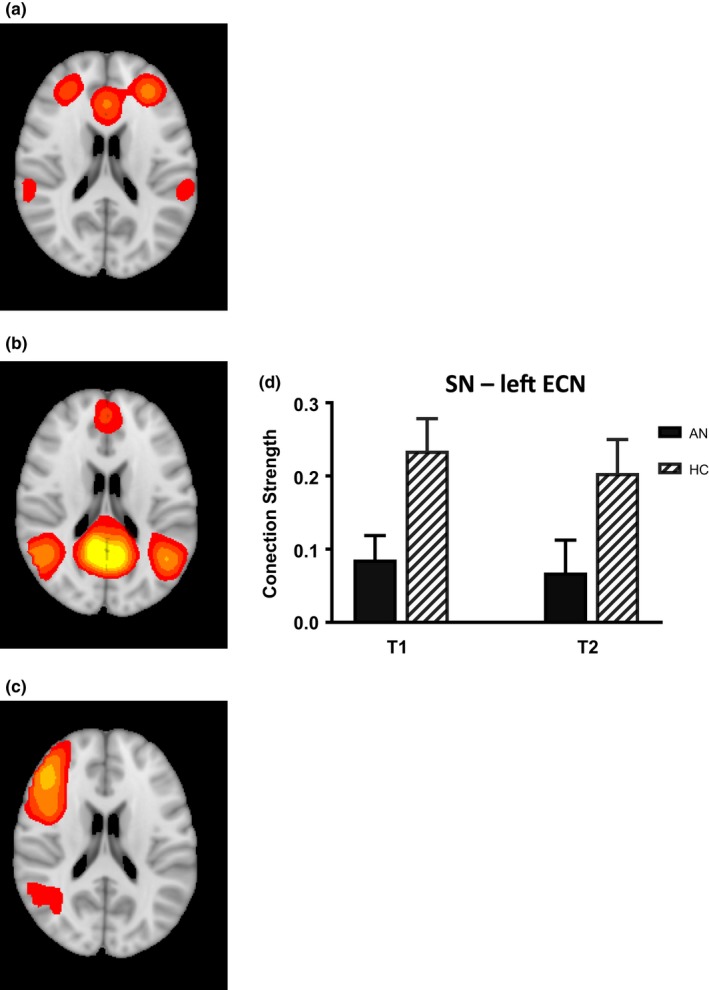
Functional connectivity analysis shows reduced connectivity between the salience network and executive control network in anorexia nervosa (AN). Resting‐state functional connectivity maps of the (a) salience network (SN) (b) default mode network (DMN) and (c) left executive control network (ECN). Bar graph (d) shows mean connectivity strength between the SN and left ECN is reduced in AN participants before and after weight restoration relative to HC (*p* = 0.001)

There was reduced cross‐network functional connectivity between the SN–left ECN in the AN group relative to HC (Group: *F*[1,45] = 11.47, *p* = 0.001) with no effect of time (*F*[1,45] = 0.54, *p* = 0.47) or interaction between time and group (*F*[1,45] = 0.08, *p* = 0.78) (Figure 2). There was no group difference in SN‐DMN or DMN‐ECN functional network connectivity at either time point. Secondary analysis of the effect of subtype on resting‐state connectivity showed reduced SN–left ECN connectivity in the AN‐R group relative to HC (Bonferroni corrected *p = *0.03) at Time 1 and reduced SN–left ECN connectivity in the AN‐BP group relative to HC at Time 2 (Bonferroni corrected *p = *0.01). There were no other significant group differences related to subtype at either time point (Bonferroni corrected *p*'s > 0.34) or no subtype difference in changes in connectivity. There were no significant correlations between inter‐network connectivity and obsessive–compulsive symptomatology among AN (Supporting information Table S2).

## 4 DISCUSSION

This study used rs‐fcMRI to examine neural substrates of obsessive–compulsive symptomatology longitudinally in individuals with AN, before, and after weight restoration treatment, compared with HC. We first examined connectivity within the limbic CSTC loop and found increased left NAcc–left mOFC connectivity among underweight individuals with AN only. We did not find evidence of increased connectivity in the AN group relative to HC following weight restoration. Contrary to our hypothesis, we did not find an association between left NAcc–left mOFC connectivity and obsessive–compulsive symptoms among individuals with AN. Our exploratory FNC analysis showed significantly weaker inter‐network connectivity between the SN and left ECN that persisted following weight restoration treatment.

Clinically, individuals with AN showed improvement in eating disorder, depressive, and obsessive–compulsive symptoms following weight restoration. These results are consistent with existing data showing improvement, but not normalization, of psychological variables with nutritional rehabilitation (Attia, Haiman, Timothy Walsh, & Flater, 1998; Mattar, Thié Baud, Huas, Cebula, & Godart, 2012; Sysko, Walsh, Schebendach, & Wilson, 2005). We did not observe improvement in ruminative thinking among participants with AN, consistent with the conception of rumination as a stable individual trait (Smith & Alloy, 2009). Rumination has rarely been examined in individuals with AN (Fürtjes et al., 2018; Rawal, Park, & Williams, 2010; Smith, Mason, & Lavender, 2018; Startup et al., 2013), and only one other study that we are aware of has examined rumination longitudinally in AN (Fürtjes et al., 2018). In depressive disorders, rumination has been associated with both the onset and maintenance of illness (Nolen‐Hoeksema, 2000; Nolen‐Hoeksema, Larson, & Grayson, 1999; Nolen‐Hoeksema, Morrow, & Fredrickson, 1993; Watkins, 2008) and has been considered as a target in the treatment of chronic depression (Watkins et al., 2011). Further research is warranted to investigate whether a ruminative thinking style is related to the persistence of AN.

The seed‐based analysis of the limbic CSTC loop (NAcc‐OFC) showed increased left NAcc–left mOFC connectivity among AN relative to HC at Time 1 only. This finding is consistent with results from Cha et  al’ multimodal neuroimaging study in AN (Cha et al., 2016) who similarly observed increased limbic CSTC connectivity at Time 1 and no group difference at Time 2. Notably, diffusion tensor imaging (DTI) results in the study by Cha et  al showed increased white matter structural connectivity in the same circuit which persisted following weight restoration (Cha et al., 2016). Taken together with our results, which also show a change in resting‐state connectivity in both patient and HC groups with time, the resting‐state functional connectivity findings appear to be less stable than the DTI results. While our MRI pulse sequences aimed to optimize measurement of the OFC, it may be that this circuit is particularly challenging to study with functional connectivity methods because of OFC signal dropout during echo‐planar imaging due to proximity to the frontal and maxillary sinuses. The FNC results, discussed below, are less vulnerable to this methodological limitation. Finally, although we observed altered NAcc‐OFC connectivity in underweight individuals with AN relative to HC, we did not find an association with obsessive–compulsive symptomatology. This result is in contrast to evidence in the OCD literature of an association between frontostriatal resting‐state connectivity and obsessive–compulsive symptom severity (Posner et al., 2014) and suggests that despite phenomenological similarities between AN and OCD, the neural underpinnings of obsessive–compulsive symptoms in the two disorders may differ.

Analysis of the effect of subtype on NAcc–OFC resting‐state connectivity showed increased left NAcc–left mOFC connectivity in the AN‐BP group relative to HC at Time 1 and no significant difference between AN‐BP and AN‐R or between AN‐R and HC at either time point. Subtypes effects have not been systematically assessed in prior research. Some studies have not reported subtype analyses (Biezonski et al., 2016; Favaro et al., 2013; Phillipou et al., 2016) and other studies only included AN‐R (Haynos et al., 2018). One study had unbalanced numbers in each subtype group which limited between‐group comparisons (Boehm et al., 2016). Cha et  al included both AN‐R and AN‐BP and found no effect of subtype on imaging results (Cha et al., 2016). Our patient sample was slightly larger, which may have allowed us to detect a group difference that was not evident with a smaller group.

This is the third study that we are aware of to find NAcc‐ prefrontal cortex rs‐fcMRI differences between individuals with AN and HC. The direction of effect differs across studies. Our patient sample was unmedicated and included individuals with both AN subtypes, whereas the study by Haynos et al. (2018) permitted psychotropic medications and studied individuals with AN‐R only. As frontal activation has been shown to change with psychotropics (Posner et al., 2011), medication status may influence frontostriatal circuit connectivity results. However, while the discrepancy in our results is likely due in part to these methodological differences, the divergence in our findings also mirrors results from the broader fMRI literature in AN in which some studies have found evidence of increased neural activity within the frontostriatal system (Cowdrey, Park, Harmer, & McCabe, 2011; Frank et al., 2012) while others have found the opposite (Brooks et al., 2011; Scaife et al., 2017; Wagner et al., 2007). Moving forward, it will be important to discern whether the heterogeneity in results stems from variability in methodology or reflects an underlying complexity of frontostriatal circuitry in AN.

The FNC analysis demonstrated reduced connectivity between the SN and left ECN in the AN group. Reduced SN–left ECN connectivity among AN persisted with weight restoration, suggesting that the observed disturbances in cross‐network connectivity are not limited to the state of acute malnourishment. Boehm et al. (2016) measured FNC between the SN, DMN, and FPN (analogous to ECN) in a group who had recovered from AN compared with HC and did not find a significant group difference in cross‐network interactions. More work is needed to better understand the relationship between stage of illness and triple network functional connectivity, and longitudinal studies are particularly vital as individuals who recover from AN may differ in important ways from those with more persistent illness.

We did not find an association between triple network connectivity and measures of obsessive–compulsive symptoms or rumination. Given the proposed roles of the SN and ECN in attention, working memory, and goal‐directed decision making (Koechlin & Summerfield, 2007; Menon & Uddin, 2010; Müller & Knight, 2006), it may be that impaired connectivity between the SN and ECN in AN is related to alterations in attention and cognition that were not tested in this study. Future studies testing other dimensions of attention and cognitive flexibility which have been shown to be impaired in AN, such as set‐shifting (Tchanturia et al., 2004; Tchanturia, Morris, Surguladze, & Treasure, 2002) and inhibition of attention (Weinbach, Sher, Lock, & Henik, 2018), may help characterize how disturbances in SN–ECN connectivity relate to cognitive symptoms in AN.

This study has notable strengths. First, the patients with AN were not taking psychotropic medication. The full effects of psychotropic medications on the brain, and especially on functional connectivity, are unknown. However, as functional connectivity within large neural circuits has been shown to change with psychotropics (Posner et al., 2011), medication status may have a substantial influence upon frontostriatal circuit connectivity results. Second, fMRI testing occurred at two time points, before and after weight restoration in the same participants. As the effect of stage of illness on brain architecture and connectivity is not fully established, this longitudinal analysis provides information about the stability of neural network changes in AN. As there may be important differences in the neurobiology of individuals who recover, a longitudinal approach can provide different information than cross‐sectional studies. A potential limitation of this study is that estimated IQ scores were not available for individuals under 16 years old. However, a sensitivity analysis of our imaging results in which we removed the six participants under the age of 16 did not meaningfully alter results. Studying adolescents also raises unique challenges due to the influence of hormones on brain development. This study did not include assessments of puberty, which may be useful covariates in future studies of adolescents with AN. Imaging‐related limitations include the possibility that participants fell asleep during resting‐state scans, despite being instructed before beginning fMRI procedures to rest with their eyes closed but to remain awake, and the difficulty in scanning the OFC given this region's vulnerability to OFC signal dropout. Finally, the FNC analysis was exploratory and should be considered as useful in generating new hypotheses about the relationship of altered triple network FNC to the underlying neurobiology of AN.

This study found evidence of increased left NAcc–left mOFC connectivity among underweight AN that was not observed following weight restoration. Unlike in OCD, we did not find an association between altered NAcc‐OFC connectivity and obsessive–compulsive symptoms in AN. Our exploratory investigation of the triple network model revealed evidence of disturbed connectivity in AN across large‐scale neural networks which persisted following weight restoration. Taken together, these results suggest that while there is clear value in probing the neurobiology of illness‐specific phenomena to understand the neurobiology of AN, advances in psychiatry more broadly may come through probing complex inter‐network relationships.

## CONFLICT OF INTEREST

Dr. Steinglass receives royalties from UpToDate.
