# Supplementary material for: Resting‐state connectivity within and across neural circuits in anorexia nervosa
Source: Brain Behav. 2018 Dec 27;9(1):e01205. doi: 10.1002/brb3.1205 (PMC6373651; doi:10.1002/brb3.1205)
Supplement: Supplementary file 1 [file BRB3-9-e01205-s001.docx]

Supplemental Material for

**Resting State Connectivity Within and Across Neural Circuits in Anorexia Nervosa**

Blair Uniacke, Yun Wang, Dominik Biezonski, Tamara Sussman, Seonjoo Lee,

Jonathan Posner, Joanna Steinglass

**Functional connectivity and clinical measures**

**Methods**

Partial correlations were performed to measure associations between fMRI results, clinical measures and weight slope, controlling for age and estimated IQ scores (demeaned).

**Results**

Seed-based connectivity (left NAcc- left mOFC): There were no significant associations between left NAcc - left mOFC connectivity and obsessive compulsive symptoms or rumination (Table S1) or weight slope (*r* =0.27, *p* = 0.26).

| **Table S1**. Associations between left NAcc – left mOFC connectivity and clinical measures | | | | |
| --- | --- | --- | --- | --- |
|  | Time 1 | | Time 2 | |
|  | *r* | *p* | *r* | *p* |
| OCI-R | 0.22 | 0.32 | 0.01 | 0.96 |
| YBC-EDS | 0.18 | 0.42 | -0.01 | 0.97 |
| RRS | -0.33 | 0.12 | 0.10 | 0.71 |

FNC: There were no significant correlations between SN – left ECN connectivity and obsessive compulsive symptoms or rumination (Table S2) or weight slope (*r* = -0.33, *p* = 0.22).

| **Table S2**. Associations between SN – left ECN connectivity and clinical measures | | | | |
| --- | --- | --- | --- | --- |
|  | Time 1 | | Time 2 | |
|  | *r* | *p* | *r* | *p* |
| OCI-R | -0.05 | 0.85 | 0.29 | 0.28 |
| YBC-EDS | -0.15 | 0.58 | 0.17 | 0.54 |
| RRS | -0.05 | 0.88 | 0.24 | 0.37 |

**Supplemental Figure 1**: Head Motion Group Comparisons

Average FDs before motion correction (frame-wise displacement) for each group were compared using repeated measures ANOVA. There was no effect of time (*F*(1,48)=1.22, *p*=0.27), or group (*F*(1,48) <0.0001, *p*=0.98), and no interaction between time and group (*F*(1,48)=1.87, *p*=0.18).

**Supplemental Figure 2**: Head Motion Correction Comparisons between FIX and CompCor Method

We also compared the performance of the two different denoising methods (CompCor and ICA+FIX) used in our paper with average FD measurement. The average FD after FIX is statistically better than CompCor method (*p*<0.001).

**Table S3**: Network Identification

We calculated four different metrics (overlay, dice, Jaccard, and PearsonsSCC) using the ICN Atlas toolbox (Kozak, van Graan, Chaudhary, Szabo, & Lemieux, 2017) to quantify how well our group ICA derived networks, including the DMN, and the right and left executive control networks, correspond to Smith’s resting-state network 2009 (Smith et al., 2009) atlas template. To define the salience network, we used the BrainMap20 from Laird et al (Laird et al., 2011).

**References**

Kozak, L.R., van Graan, L.A., Chaudhary, U.J., Szabo, A.G., Lemieux, L. (2017). ICN_Atlas: Automated

description and quantification of functional MRI activation patterns in the framework of intrinsic connectivity networks. *Neuroimage,* 163, 319-341. Doi:10.1016/j.neuroimage/2017.09.014

Laird, A.R., Fox, P.M., Eickhoff, S.B., Turner, J.A., Ray, K.L., McKay, D.R., …Fox, P.T. (2011). Behavioral

interpretations of intrinsic connectivity networks*. Journal of Cognitive Neuroscience*, 23(12), 4022-4037.

Smith, S.M., Fox, P.T., Miller, K.L., Glahn, D.C., Fox, P.M., Mackay, C.E., …Beckmann, C.F. (2009).

Correspondence of the brain’s functional architecture during activation and rest. *Proceedings of the National Academy of Sciences of the United States of America*, 106(31), 13040-5. <https://doi.org/10.1073/pnas.0905267106>
